# Autophagy Protects from Trastuzumab-Induced Cytotoxicity in HER2 Overexpressing Breast Tumor Spheroids

**DOI:** 10.1371/journal.pone.0137920

**Published:** 2015-09-11

**Authors:** Cristina E. Rodríguez, Sara I. Reidel, Elisa D. Bal de Kier Joffé, Maria A. Jasnis, Gabriel L. Fiszman

**Affiliations:** 1 Research Area, Institute of Oncology ‘Ángel H. Roffo’, University of Buenos Aires, Buenos Aires, Argentina; 2 Industrial Biotechnology Research and Development Center, National Institute of Industrial Technology, Buenos Aires, Argentina; University of Torino, ITALY

## Abstract

Multicellular tumor spheroids represent a 3D *in vitro* model that mimics solid tumor essential properties including assembly and development of extracellular matrix and nutrient, oxygen and proliferation gradients. In the present study, we analyze the impact of 3D spatial organization of HER2-overexpressing breast cancer cells on the response to Trastuzumab. We cultured human mammary adenocarcinoma cell lines as spheroids with the hanging drop method and we observed a gradient of proliferating, quiescent, hypoxic, apoptotic and autophagic cells towards the inner core. This 3D organization decreased Trastuzumab sensitivity of HER2 over-expressing cells compared to monolayer cell cultures. We did not observe apoptosis induced by Trastuzumab but found cell arrest in G0/G1 phase. Moreover, the treatment downregulated the basal apoptosis only found in tumor spheroids, by eliciting protective autophagy. We were able to increase sensitivity to Trastuzumab by autophagy inhibition, thus exposing the interaction between apoptosis and autophagy. We confirmed this result by developing a resistant cell line that was more sensitive to autophagy inhibition than the parental BT474 cells. In summary, the development of Trastuzumab resistance relies on the balance between death and survival mechanisms, characteristic of 3D cell organization. We propose the use of spheroids to further improve the understanding of Trastuzumab antitumor activity and overcome resistance.

## Introduction

HER2 is a member of the human epidermal growth factor receptor (HER/ErbB) family of tyrosine kinases which also includes EGFR, HER3 and HER4. Human breast cancers with overexpression of HER2, occur in about 20% of patients and are associated with poor prognosis [1]. Trastuzumab (Tz, Herceptin), a humanized monoclonal antibody, binds the extracellular region of HER2 and inhibits receptor signaling via several mechanisms [2–4]. Even though treatment with Tz is the alternative choice in HER2-positive breast cancer treatment [5], only a fraction of metastatic patients respond to Tz as single agent and approximately 60% develop resistance after initial response [6,7].

Tumor microenvironment plays an important role as pro-survival factor for remaining living cells after initial chemotherapy and it is also involved in mechanisms that facilitate drug-resistance [8]. For many years, tumor resistance have been investigated using tumor cell lines grown as monolayers, but lack of correlation with clinical data suggests that 2D cultures might not reflect critical aspects of tumor growth. Cancer cells cultured as 3D spheroids represent a more useful model, since cell behavior changes considerably in a microenvironment that mimics the complex 3D organization of avascular tumor tissue *in vivo*, with differential oxygen and nutrient supply [9] and accumulation of cell metabolites from the outer to the inner spheroid regions [10]. Consequently, spheroids have a marked cellular, physiological and structural heterogeneity, relevant in cancer biology.

Autophagy is a dynamic self-catabolic cellular process that preserves cellular homeostasis by regulating degradation of misfolded proteins and of aged or damaged organelles, thus controlling cell damage [11]. The autophagic process is upregulated during periods of stress to maintain cell viability by allowing basic biomolecules and energy recycling. A link between autophagy and HER2 expression has been described in a subset of human breast carcinomas: the loss of the autophagy regulator *beclin 1* correlates with HER2 amplification while patients with *beclin 1* gene responded better to Tz alone or in combination with other drugs [12]. Many efforts have been made to analyze the effect of autophagy blockade on the response to chemotherapy, but they have mainly been focused on the tumor cells themselves. Functional autophagy in cancer should be considered as an important piece of the tumor microenvironment [13].

In the present study, we analyze the mechanisms of action and resistance development in the treatment with Tz using a model of multicellular tumor spheroids. We provide evidence that in 3D cells organization, autophagy efficiently protects breast cancer cells from the growth-inhibitory effect of Tz, and therefore, spheroids could be a more accurate model than monolayers to investigate anti-cancer drug action and anti-tumor drug resistance mechanisms.

## Materials and Methods

### Cell cultures and generation of tumor spheroids

Trastuzumab (Tz, Herceptin) was used at different concentrations (0.05–50 μg/ml); an unrelated human IgG (UNC Hemoderivados) was used as isotype control. Human mammary adenocarcinoma BT474 and MCF7 cell lines, obtained from American Tissue Culture Collection (ATCC), were grown in RPMI 1640 and DMEM-F12 respectively (Gibco, Life Technologies) supplemented with 10% fetal bovine serum (Internegocios S.A.) and gentamicine. Serial passages were carried out by treatment with 0.25% trypsin and 0.075% EDTA (Sigma).

Tz- resistant BT474 cells (BT474-MR) were obtained by continuous treatment of monolayers with Tz (10 μg/ml) during up to 6 months.

To generate spheroids, we adapted the hanging drop method [14]. Briefly, 1x10^4^ cells were seeded on the cover of 48-well plates in 20 μl drops. Covers were then inverted and incubated for 72 h until spheroids were fully formed, after which they were transferred into individual wells coated with 1.5% agarose and 500 μl complete medium. Spheroids were fed every other day by carefully aspirating 250 μl of medium and replacing it with the same volume of fresh complete medium. To evaluate Tz chronic treatment in 3D, experiments were performed when spheroids reached a diameter ≥ 550 μm, corresponding approximately to day 7. The adequate concentration of Tz was added in each media replacement, performed every other day and constantly maintained during the experiment. To evaluate spheroids growth, microphotographs were taken periodically and analyzed by Image Pro Plus software.

### Immunostaining

Spheroids, obtained as described above, were treated with 50 μg/ml Tz for 15 days. At the end of the experiment, spheroids were collected and fixed in buffered formalin for 30 min, dehydrated in alcohol gradient, embedded in paraffin and sectioned (5 μm thickness). Sections were deparaffinized in xylene, rehydrated in an alcohol gradient. Antigen retrieval was performed by heating for 30 min in citric acid buffer (pH 6.0). Sections were then blocked with PBS: 5% FBS for 1 h at room temperature and incubated with primary antibodies at 4°C, overnight. Sections were then washed with PBS and incubated for 1 h with the corresponding secondary antibody at room temperature. For immunofluorescence studies, slides were also incubated with propidium iodide (PI) for nuclear detection and visualized using a confocal microscope (Olympus fv300/bx6). Primary antibodies used were anti-Ki67 (M7240, DAKO), anti-light-chain 3B (LC3B, #2275), anti-HER2 (#2165), anti-pHER2 (Y1248, #2247) (Cell Signaling), anti-HIF-1α (sc-13515) and anti-p27 (sc-528) (Santa Cruz Biotechnology). Secondary antibodies used were anti-rabbit IgG AlexaFluor 488 (Invitrogen) and anti-rabbit IgG HRP conjugate (Millipore). All antibodies were used at the appropriated dilution suggested by manufacturer. Sections were also stained with hematoxylin-eosin (H&E) for histology analysis. To detect autophagic flux, cells were treated with Tz 1μg/ml or control IgG for 24 h, and Bafilomycin A1 (Santa Cruz Biotechnology) at 5 nM was added 90 min before the end of the experiment. Cells were then fixed and stained with anti-LC3 antibody as mentioned above.

### Evaluation of apoptosis

Apoptosis was evaluated in 2D as well as in 3D cultures treated with Tz (1 μg/ml or 50 μg/ml respectively). Spheroids were disaggregated with Trypsin-EDTA for 15 min at 37°C and monolayers were harvested as mentioned above. Cells were stained with Annexin V-FITC and propidium iodide (PI) for 15 min at room temperature according to manufacturer’s instructions (Invitrogen). Fluorescence was detected using a flow cytometer (Partec, Pas III model) and Flomax software. Data were analyzed with the WinMDI software. As reported elsewhere [15], early apoptosis is related to cells that are annexin V positive but remain impenetrable to PI, whereas late apoptotic cells are both annexin V and PI positive. Viable cells are negative for annexin V and PI stain.

### Cell cycle analysis

2D and 3D cultures, treated and harvested as mentioned above, were fixed in 70% ethanol for 2 h. DNA was stained with a solution containing 0.1% v/v Triton X-100, propidium iodide (25 μg/ml) and RNase A (1 mg/ ml, Boehringer Mannheim GmbH). Cell cycle was studied by flow cytometry and data were analyzed with the Cyflogic software (CyFlo Ltd, Finland).

### Western blot

Protein extracts were prepared by homogenizing monolayers or spheroids in RIPA buffer (20 mM Tris-HCl, pH 7.5, 150 mM NaCl, 0.1% de SDS, 1% NP40, 0.5% deoxycholate and 1 mM EDTA) containing protease and phosphatase inhibitor cocktails (Calbiochem). Protein concentrations were measured using the Bradford method. Primary antibodies anti-Akt 1/2/3 (H-136, sc-8312), anti-phospho Akt 1/2/3 (Ser 473, sc-7985), anti-β-actin (sc-47778) (Santa Cruz Biotechnology) and anti-LC3B (#2275, Cell Signaling) were used, at the appropriated dilution suggested by manufacturer. Immunoreactive bands were detected by enhance chemiluminescence (ECL, Amersham Biosciences).

### Cell viability

Cells cultured in 96-well plates were incubated with increasing concentrations of Tz (0.08–50 μg/ml) or IgG (50 μg/ml). Cell viability was determined using the colorimetric MTS assay (Cell Titer ^96^ non radioactive cell proliferation assay kit, Promega). Briefly, after 5 days treatment with Tz, medium was replaced with MTS/PMS solution (1 ml MTS: 20 μl PMS) in fresh medium. Cells were then incubated for 2 h at 37°C in a humidified atmosphere and absorbance of cell-free supernatants was measured at 490 nm using the Multiscan Ascent microplate reader.

Since autophagy could affect cell metabolism, cell viability after autophagy inhibition was also analyzed by the Trypan blue exclusion method. Briefly, cells were plated in 12-well plates at a density of 20x10^3^ cells/ cm^2^ and treated simultaneously with 1 μg/ml Tz and 1 mM 3-methyl adenine (Santa Cruz Biotechnology). Drugs and medium were left unchanged for 3 days, when cell viability was determined.

### Statistical analysis

All experiments were done in triplicate and were repeated at least twice. Data were expressed as mean values +/- SE or SD and analyzed by Student’s test or ANOVA followed by Bonferroni’s posttest analysis to compare differences between groups. GraphPad Prism V software was used and data were considered statistically significant when p<0.05.

## Results

### The 3D architecture and growth kinetics of tumor spheroids

**Fig 1:**
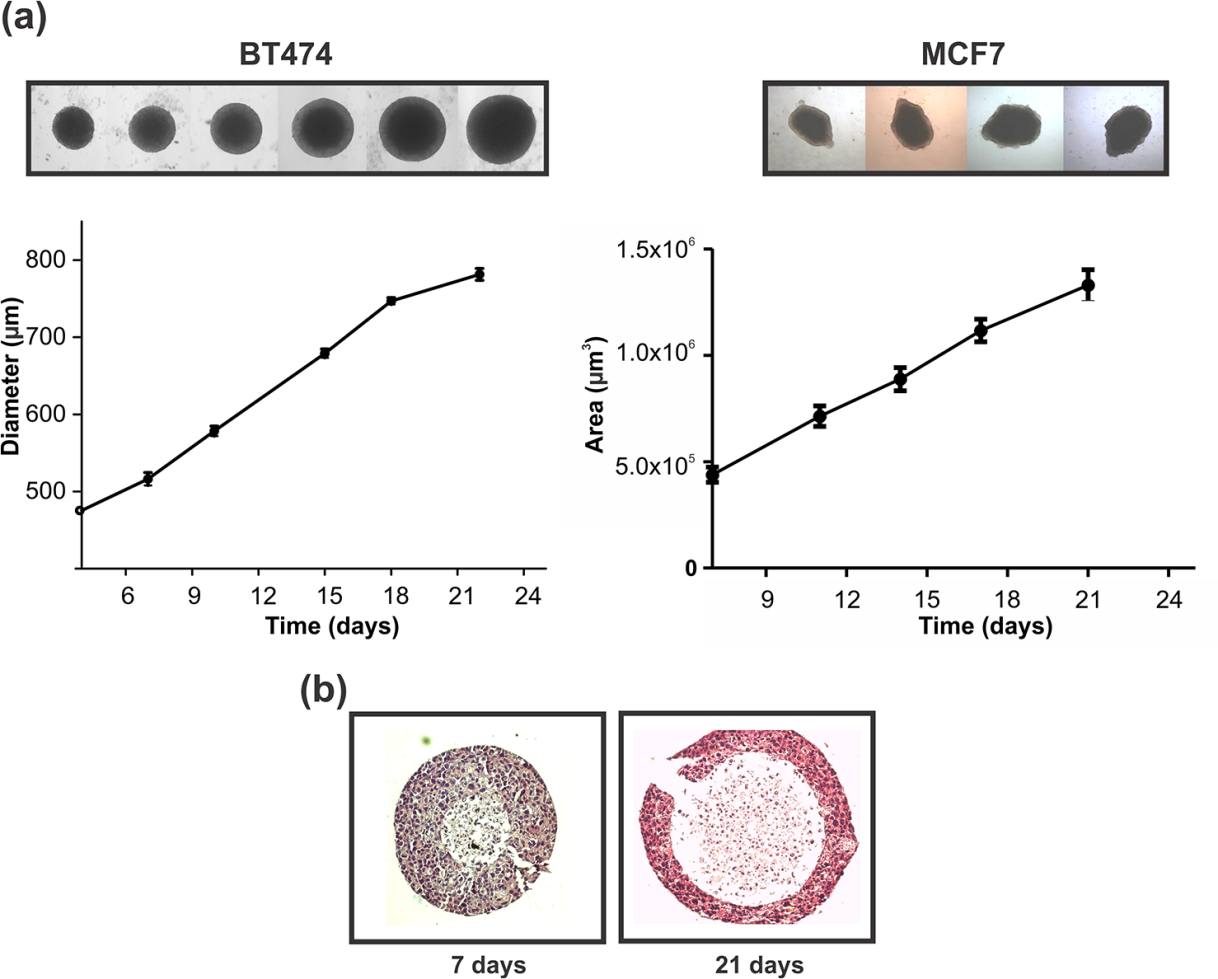
Cells growth in 3D. (a) Kinetics of BT474 and MCF7 spheroids growth, n = 6. Upper insert corresponds to representative photographs taken to the same spheroid along time (40X). (b) Hematoxylin-Eosin staining of BT474 spheroid at 7 and 21 days growth showing the necrotic cores and the peripheral rims of viable cells (200X).

Breast cancer cells BT474, with HER2-overexpression and MCF7 cells, with low levels of HER2 expression were selected to generate spheroids due to their capacity to grow in 3D. Since BT474 cells generated regular spheres, we considered diameters as a measure of spheroids growth; on the other hand, as MCF7 spheroids grew irregularly, we chose the area as a measure of growth. Both spheroid types exhibited a linear growth up to day 22 (Fig 1A).

Seven days after initial seeding, BT474 spheroids showed an average diameter of ~550 μm, and displayed a small central necrotic core surrounded by a rim of living cells. At day 20, spheroids reached an average diameter ≥ 750 μm, associated with changes in their 3D architecture, such as a bigger necrotic core and a thinner peripheral border of viable cells (Fig 1B); beyond 25–30 days, spheroids started to distort. These observations led us to determine the proliferative state of spheroid’s cells by flow cytometry. Our experiments showed that during spheroids growth, no changes in the percentage of cells in G0/G1 phase were detected, but an increase in the proliferative population (S phase) up to day 14 and an arrest in G2/M along culture (data not shown).

### Trastuzumab induces arrest at G0/G1 phase and reduces late apoptosis

**Fig 2:**
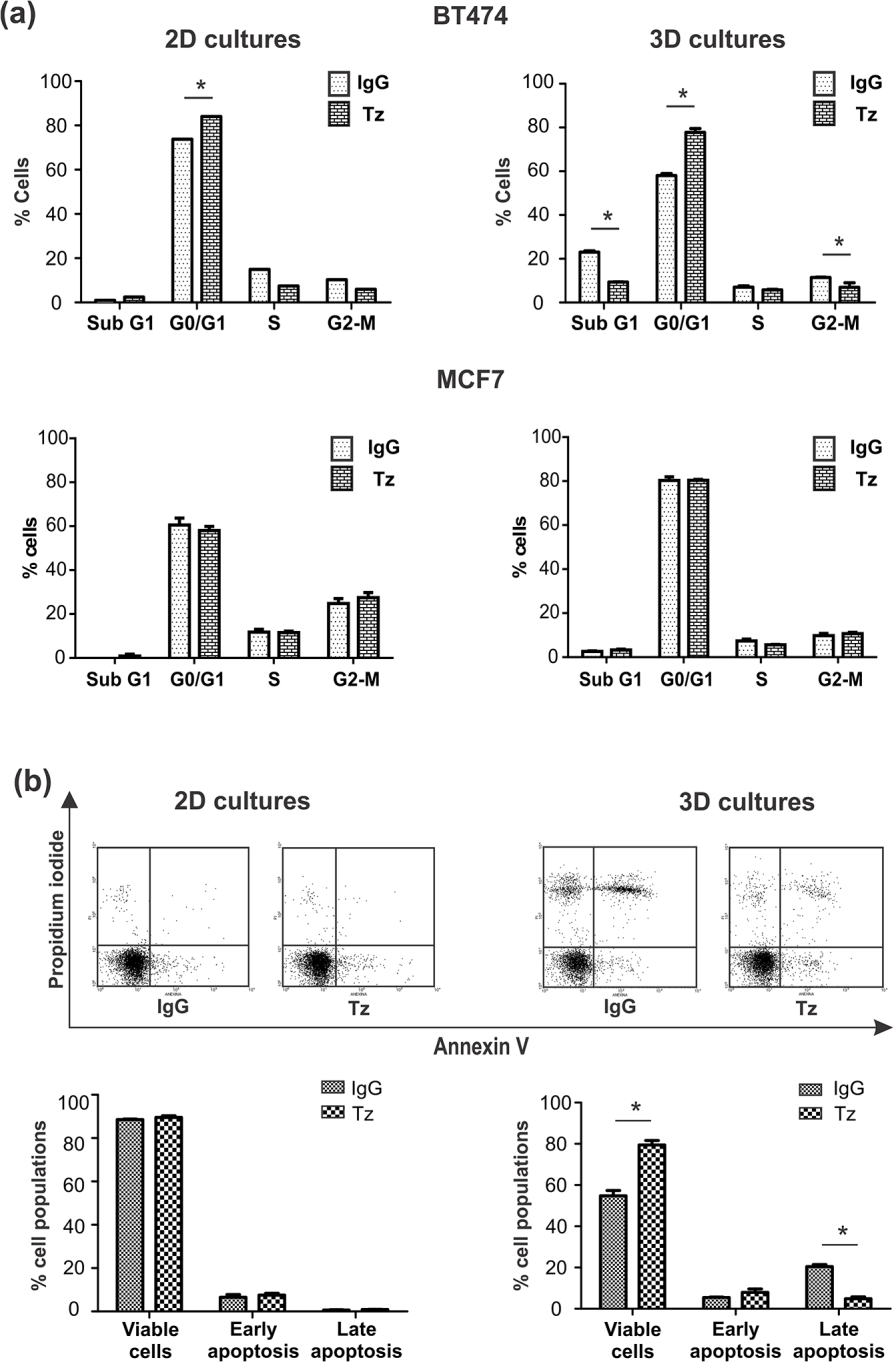
Effect of Tz on cell cycle and apoptosis. BT474 and MCF7 cells were treated for 24 h with 1 μg/ml Tz (2D), 50 μg/ml Tz (3D) or control IgG, and were analyzed by flow cytometry. (a) Quantification of cell cycle distribution analyzed with propidium iodide; (b) Apoptosis analysis with Annexin V/ PI and quantification of BT474 cells. Triplicate cultures were run per group, and each experiment was repeated three times (*p<0.05).

We analyzed the effect of the monoclonal antibody Trastuzumab (Tz) on the cell cycle distribution of BT474 and MCF7 cells, cultured as spheroids and as monolayers. Our results showed that 24 h treatment with Tz modulates the cell cycle of BT474 cells both in 2D and 3D cultures and has no significant effect on the cell cycle of MCF7 cells (Fig 2A). In BT474, Tz induced an increase in G0/G1 cell population by 34% in spheroids and 13% in monolayers, compared to IgG control treatment. Also, cells in G2/M phase were reduced with Tz treatment in both culture conditions. Unlike 2D cultures, BT474 spheroids showed basal apoptosis (SubG1 phase), which decreased about 50% after Tz treatment.

To further analyze the effect of Tz on cell death observed in sub G1 subpopulation, we used the Annexin V/propidium iodide (PI) assay, which allows us to differentiate single stained Annexin V positive cells in early apoptosis and cells double stained with Annexin V and PI in late apoptosis. We found cells in late apoptosis only in 3D cultures; interestingly, 24 h treatment with Tz decreased this population (20.4% IgG vs 4.8% Tz, p<0.05) (Fig 2B). These results indicate that the effect of Tz might be mainly on preventing cell proliferation rather than on inducing cell death.

### Chronic treatment with Trastuzumab induces a resistant phenotype in 3D cultures

**Fig 3:**
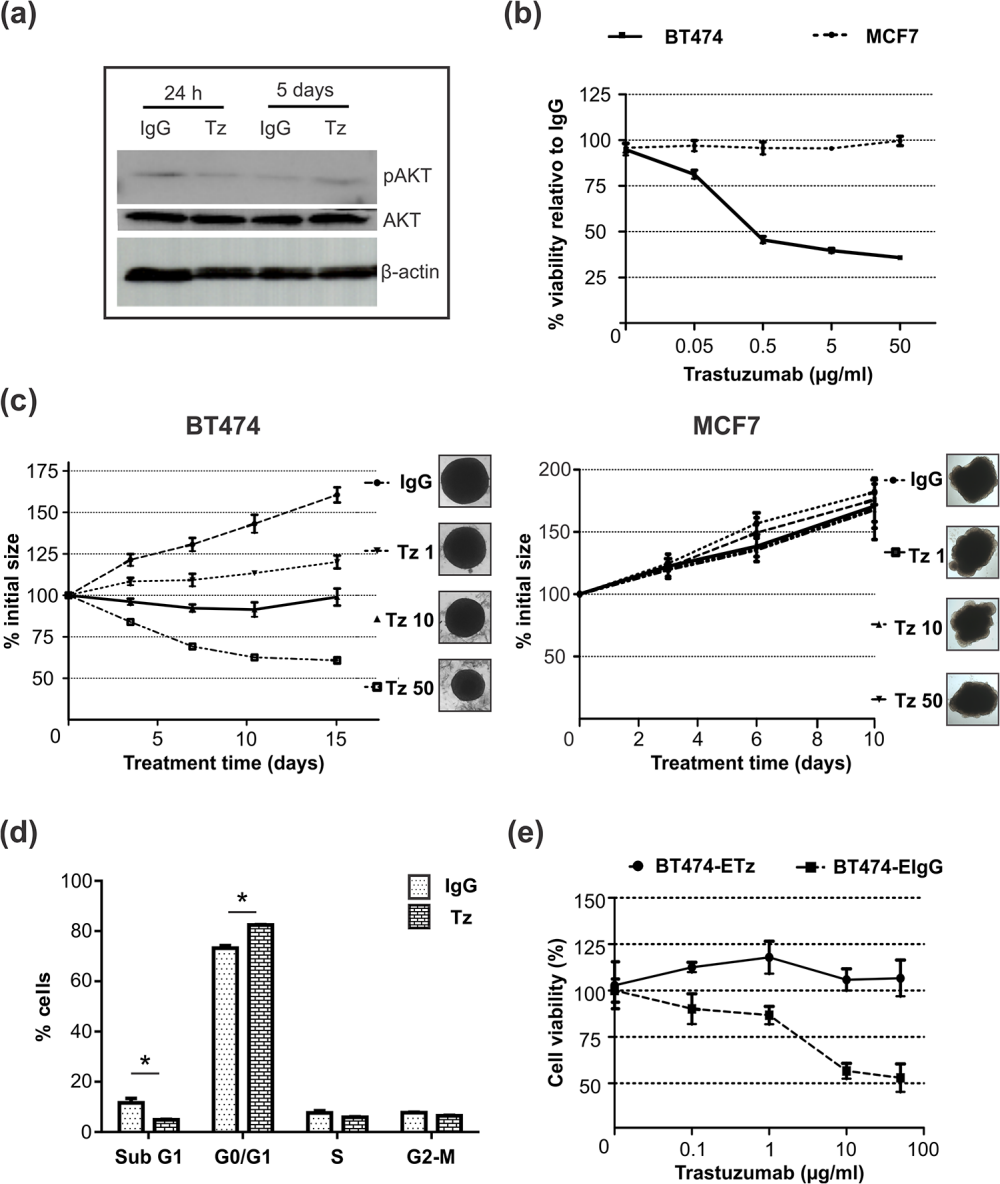
Dose-dependent effect of Tz on cells growth. (a) Western blot analysis for AkT, pAkT and β-actin of BT474 spheroids after 24 h or 5 days of Tz treatment. (b) Viability (MTS assay) of BT474 and MCF7 cells cultured as monolayers treated for 5 days with increasing concentrations of Tz (0.005–50 μg/ml) relative to isotype IgG control. (c) Volume was evaluated in BT474 and MCF7 spheroids chronically treated during 15 days with 50, 10 and 1 μg/ml Tz or human IgG. Each point of the curves expresses the percentage in size change respect to the initial pretreated size (considered 100%), and represents the mean ± SD (n = 6). Inserts (right) correspond to 15 days-treated spheroids (40X). (d) BT474 spheroids chronically treated were analyzed by flow cytometry and the quantification of cell cycle distribution with propidium iodide staining is shown. Triplicate cultures were run per group, and each experiment was repeated twice (*p<0.05). (e) Cells obtained from spheroids chronically treated with Tz (BT474-ETz) or IgG (BT474-EIgG) were cultured as monolayers and treated with increasing concentrations of Tz (0.01–50 μg/ml).

The PI3K/Akt pathway regulates cell survival and aberrations in this pathway have been involved in Tz-resistance [16]. In BT474 spheroids, we found downregulation of pAkt after 24h Tz treatment by Western Blot; however, after a continuous exposure to Tz of 5 days, this modulation was no longer detectable (Fig 3A). According to these observations, it was reasonable to hypothesize that the mechanisms involved in Tz resistance may appear with a chronic treatment; we therefore investigated the effect of a longer exposure to Tz. We used BT474 as well as MCF7 cells; 2D cultures were continuously treated with Tz for 5 days and 3D cultures were maintained in the continuous presence of Tz for 15 days. Our results showed that BT474 cells grown in 3D organization were less sensitive to Tz than those growing in 2D. In monolayers, a decrease of 24% in cell viability was detected using 0.1 μg/ml Tz, reaching the maximum effect at a dose of 1 μg/ml, when a reduction of 60% in cell viability was detected. Higher concentrations did not further increase cytotoxicity (Fig 3B). As shown in Fig 3C, throughout the 15 days treatment, control IgG spheroids had a growth curve similar to untreated spheroids (Fig 1A). At the end of experiment, Tz used at a concentration of 50 μg/ml, was able to reduce the initial spheroids size by 40%; however, when treated with 10 μg/ml Tz, spheroids remained with their initial size. At an even 10-fold lower concentration (1 μg/ml), Tz exerted a minor regulation on spheroids size, but a slower growth of Tz treated spheroids could still be detected, compared to controls (Fig 3C). These dose dependent effects were fully reversed after Tz withdrawal (data not shown). When cell viability in chronically treated spheroids was analyzed by Trypan blue exclusion method, the % of dead cells did not significantly change among the different concentrations (30.6% Tz 1 μg/ml, 22.7 Tz 10 μg/ml, 20.8 Tz 50 μg/ml and 21.5% IgG). Tz had no effect on MCF7 cell growth, either in 2D or in 3D culture conditions (Fig 3B and 3C).

We next analyzed Tz effect on cell cycle distribution of BT474 spheroids. As we observed before for 24 h treatment, chronic treatment with Tz induced cell cycle arrest in G0/G1 phase and downregulated SubG1 subpopulation (Fig 3D).

To further examine the phenotype of the remaining cells in the BT474 spheroids chronically treated with Tz, spheroids were disaggregated with trypsin-EDTA and single cells were seeded to grow as monolayeres. We evaluated their sensitivity to Tz and found that cells derived from Tz-treated spheroids (BT474-ETz) were fully resistant to the antibody, even at a concentration as high as 50 μg/ml, while cells from control spheroids (BT474-EIgG) behaved as Tz sensitive cells (Fig 3E).

**Fig 4:**
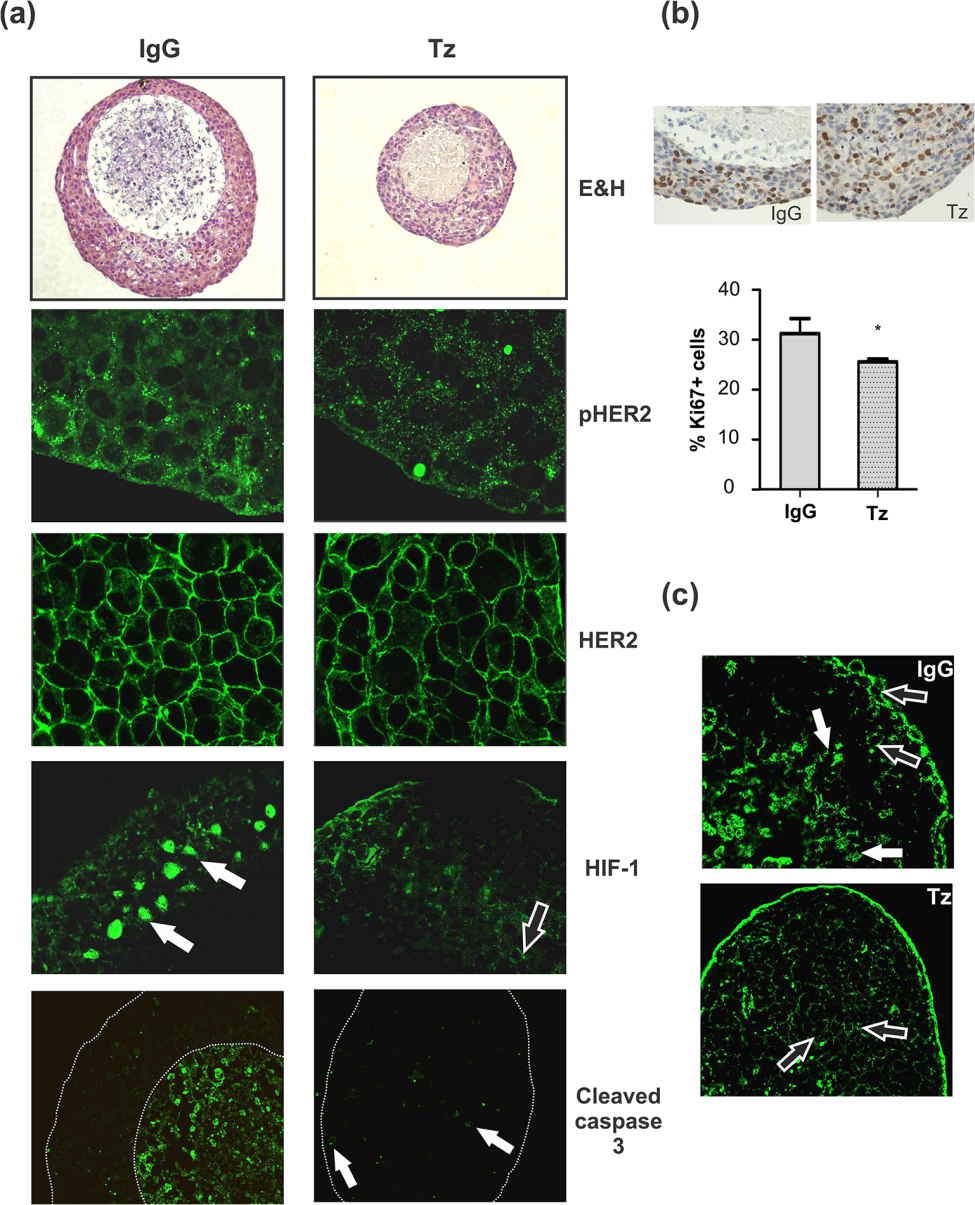
Study of spheroid subpopulations. Spheroids were treated with 50 μg/ml Tz or control IgG for 15 days. (a) H&E (200x); immunofluorescence for pHER2 and HER2, HIF-1α (600x) and cleaved caspase 3 (400x). (b) Immunohistochemistry for Ki67 and quantification of Ki67+ cells (*p<0.05). (c) Confocal analysis of p27 (200x). Filled white arrows correspond to nuclear staining and black filled arrows correspond to cytoplasmatic staining.

In order to study the molecular bases of Tz- resistance, we performed immunostaining assays in paraffin-embedded chronically treated spheroids, which allowed us to distinguish cell phenotypes based on their localization in the 3D context. We found that cell architecture of tumor spheroids changed through time in the presence of Tz, displaying a significant reduction in the size of the necrotic core after 15 days (Fig 4A). We investigated whether antitumor activity of Tz could be associated with changes in HER2 protein expression. While we did not find modulation in the receptor expression, a decrease in HER2 phosphorylation (pHER2) was detected. As hypoxia is known to play a main role in cells sensitivity to different antitumor therapies [17], we evaluated the expression of the hypoxia inducible factor 1 (HIF-1α) and its modulation by Tz. While in control spheroids HIF-1α was localized primarily in the nuclei of cells surrounding the central core, it changed its subcellular localization upon Tz addition, being found only in the cytoplasm of cells (Fig 4A). As for the expression of the apoptotis marker cleaved caspase 3, it was only detected in the central core of control spheroids, but upon Tz addition, it could not be further detected (Fig 4A).

The proliferative capacity of the peripheral viable cells in spheroids was lower than the same cell population in control spheroids, quantified as percentage of Ki67 positive cells (Fig 4B). We next studied the expression of p27, a negative regulator of the cell cycle. It was found in the cytoplasm of cells at the periphery and in the nuclei of cells located towards the central core of control spheroids. After Tz treatment, p27 was only detected in the cytoplasm of cells, regardless of their spatial localization on the spheroid (Fig 4C).

### Trastuzumab increases cell autophagy

**Fig 5:**
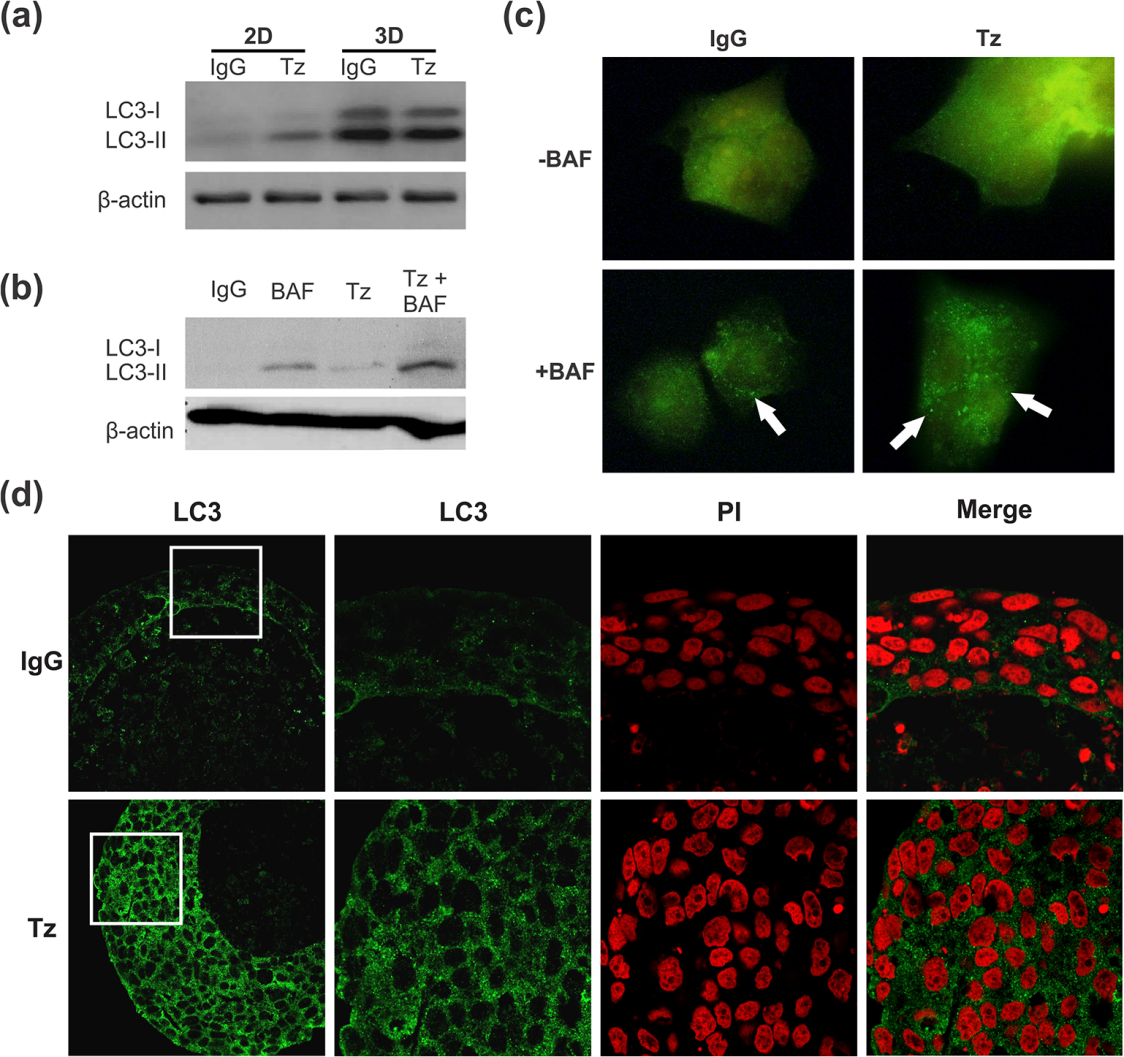
Autophagy in BT474 cells. (a) Western blot (WB) analysis of LC3 in 2D and 3D cultures after 24 h treatment with Tz. (b) Autophagic flux analyzed by WB in cell monolayers treated with Tz (1μg/ml) and 5 nM Bafilomycin A1 (BAF) (c) Cellular distribution of autophagosomes with LC3 stain. Arrows point to autophagosomes. Images show representative zones of BT474 cell monolayers (200x). (d) Immunofluorescence for LC3 (green) and Propidium iodide (PI, red) in spheroids treated for 15 days with Tz (50 μg/ml) or control IgG. Magnified images correspond to the zones limited by the white squares in the left photographs (600x).

As described above, Tz induced a decrease in the apoptotic cell subpopulation (Fig 2B). Since many reports point that autophagy could play a cytoprotective role countering anti-tumor therapies [18], we investigated if autophagy could be a mechanism involved in BT474 cell escape from apoptosis. To determine whether Tz antitumor activity might be associated with autophagy activation, the expression of the lipidated form of microtubule-associated protein light chain 3 (LC3-II) was analyzed. Compared to monolayer cell cultures, spheroids showed higher amount of LC3 (Fig 5A); addition of Tz elicited an increase in the LC3-II/LC3-I ratio in both 2D and 3D, though less evident in tumor spheroids.

To demonstrate that the accumulation of LC3-II observed after Tz treatment corresponded to increased autophagy, and was not due to lysosomal dysfunction, we used bafilomycin A1 (BAF), a V-ATPase inhibitor known to block the autophagic flux by preventing fusion between autophagosomes and lysosomes and therefore causing LC3 accumulation [19]. In the presence of BAF, we observed an increase in the levels of LC3-II/LC3-I ratio, in agreement with a blockade of lysosomal activity; treatment with Tz further increased this ratio (Fig 5B). These results suggest that the accumulation in LC3-II observed after Tz treatment was in fact due to a stimulation of the autophagic pathway. We verified these results analyzing autophagosome formation by immunofluorescence (Fig 5C). While untreated cells showed a homogenous but weak cytoplasmic LC3 staining, typical of low-level autophagosome formation, Tz-treated cells presented an increase in LC3 expression. After BAF exposure, LC3 localization dramatically changed from diffuse to a punctuate or dotted pattern associated with the autophagosome formation. We next investigated the localization of LC3 expressing cells in the spheroids. We observed a differential localization of autophagic cells, increasing in number towards the central core, thus correlating with the hypoxic population previously described. However, in spheroids chronically treated with Tz, a higher and uniform dotted expression of LC3 was found in all the living cells, which corresponds to a phenotype associated with Tz resistance (Fig 5D).

### Blockade of autophagy elicit apoptosis after Trastuzumab treatment

**Fig 6:**
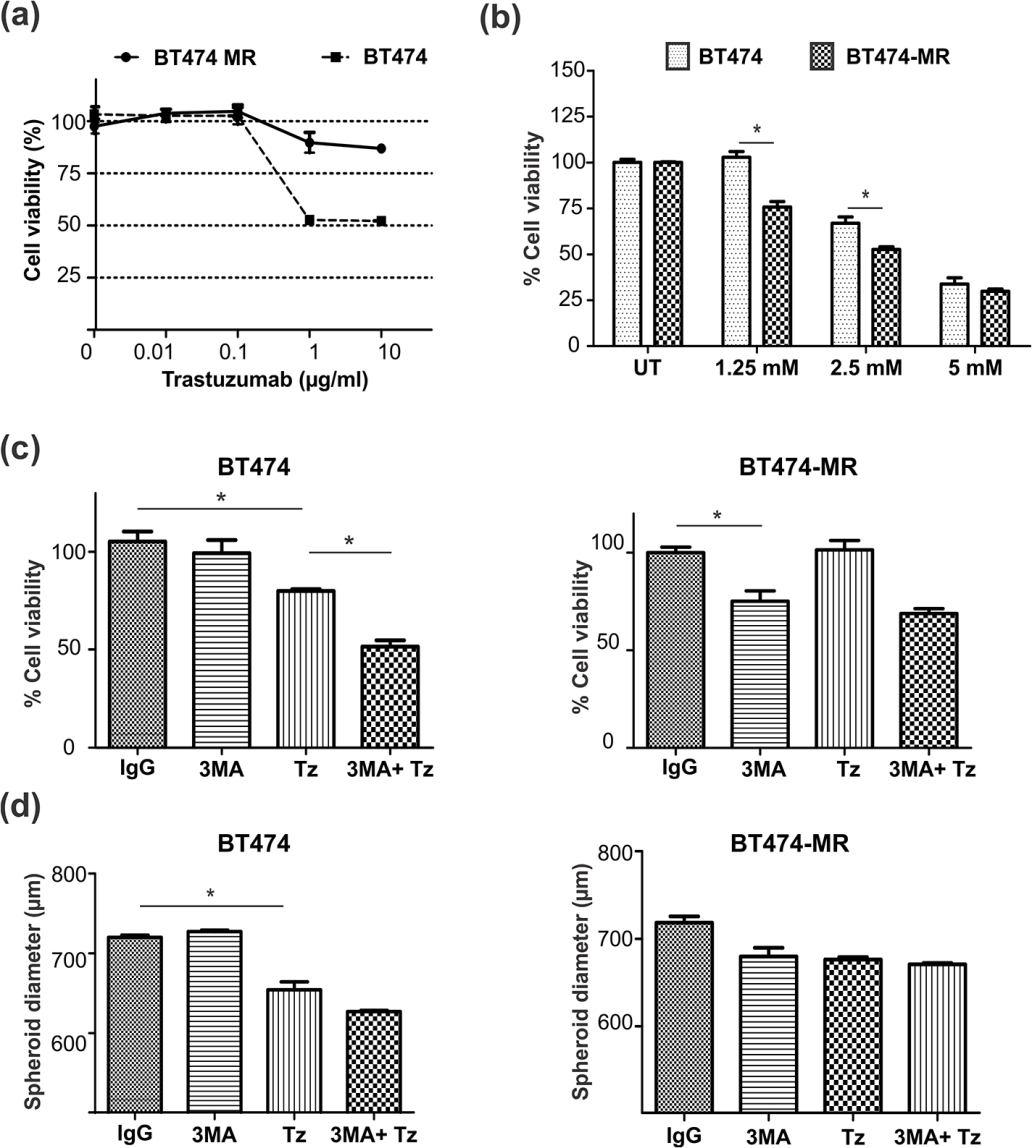
Autophagy inhibition. (a) Viability (MTS assay) of BT474 and resistant BT474-MR cells cultured as monolayers treated for 5 days with increasing concentrations of Tz relative to isotype IgG control; (b) Viability of BT474 and BT474 MR treated for 5 days with increasing concentrations of 3-MA. (c) Viability of BT474 and BT474-MR cells cultured in 2D treated with Tz (1 μg/ml), 3-MA (1 mM), or their combination for 3 days. (d) Diameter of BT474 and BT474-MR spheroids (n = 6) treated with Tz (50 μg/ml), 3-MA (1 mM) or their combination for 6 days. Each experiment was repeated at least three times (*p<0.05).

To analyze whether Tz-induced autophagy was involved in the development of acquired resistance, we generated a Tz resistant BT474 cell line, by culturing parental cells in monolayers in the presence of 10 μg/ml Tz for over 6 months; established resistant cell line was named BT474-MR (Fig 6A). We studied cell viability in both parental BT474 and resistant BT474-MR cell lines after blocking autophagy with 3-methyladenine (3-MA), which inhibits the formation of the pre-autophagosomal structures. We first detected that 3-MA was *per se* cytotoxic in a dose dependent manner. Tz resistant BT474-MR cells were more sensitive to autophagy inhibition than parental cells (Fig 6B). These results suggest that in the acquisition of resistance to Tz along time, a survival mechanism associated with increased autophagy is involved.

As 3-MA induced cell death per se. we next analyzed the effect of Tz in the presence of a non cytotoxic concentration of 3MA. In 2D cultures, this combination induced a significant increase in BT474 cell cytotoxicity compared with Tz alone (50% vs 25% respectively, p<0.05); this increase in cell death was not observed on BT474-MR cells (Fig 6C). To investigate if 3D organization modulated autophagy and thus Tz sensitivity, we measured BT474 and BT474-MR spheroids diameter after 6 days treatment. In BT474 spheroids, we did not find a cytotoxic effect of 3-MA alone but the combination of 3-MA with Tz resulted in a decrease in spheroids size compared to Tz alone (Fig 6D). On the contrary, BT474-MR spheroids size was not affected by any treatment. These results suggest that 3D organization confers an increased protection against Tz cytotoxicity in an environment with inhibited autophagy. MCF7 cell viability did not change after autophagy inhibition in either 2D or 3D cultures (data not shown).

**Fig 7:**
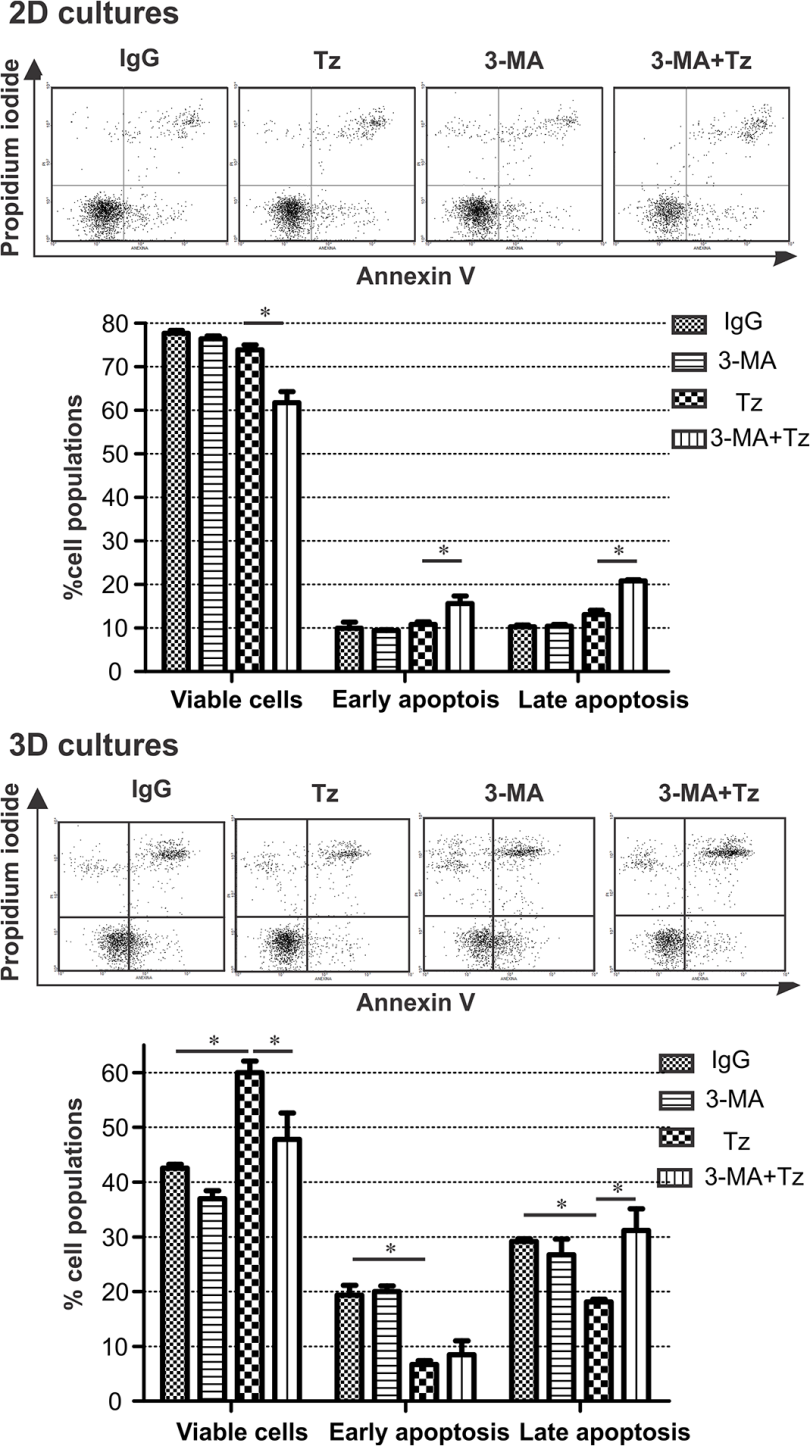
Apoptosis of cells cultured in 2D and 3D after 6 h treatment with Tz, 3-MA or their combination. Representative Annexin V/ Propidium iodide graphs are shown. Triplicate cultures were run per group, and the experiment was repeated three times (*p<0.05).

Experimental evidence suggests the existence of common links between apoptosis and autophagy in breast cancer cells [20]. We therefore investigated whether Tz-induced autophagy could be one of the mechanisms involved in rescuing cells from apoptosis as we had previously observed (Fig 2). To this end, we assessed apoptosis induction after 6h Tz treatment with 1h pre-incubation with 3-MA (Fig 7). In agreement with the results obtained for cell viability, 3-MA alone did not induce apoptosis in any culture condition. In 3D organization, the combination of Tz with 3MA completely reversed the decrease in late apoptosis induced by Tz alone; in 2D cultures, the same combination enhanced the sensitivity to Tz, determined by an increase in 30% cell apoptosis, (p<0.05). Altogether, these results point to a key role of autophagy in the inhibition of apoptosis exerted by Tz.

## Discussion

We here describe the effect of Trastuzumab (Tz) on a three-dimensional organization of HER2+ overexpressing cells. For the first time, we demonstrate that Tz decreases the apoptotic population in tumor spheroids of BT474 human HER2+ cells, effect completely reversed by autophagy inhibition.

Solid tumors contain heterogeneous populations of cells including proliferating, quiescent and necrotic. Spheroids serve as an intermediary model between the oversimplified 2D cultures and the high complexity of in vivo tumors.

Our experiments were performed with BT474 and MCF7 spheroids that presented a defined heterogeneity in cell subpopulations, such as peripheral viable and proliferating cells, a hypoxic non-proliferating intermediate cell layer and a central necrotic core (Fig 1).

Upon Tz treatment, we observed that BT474 cells were sensitive to the cytotoxic effect, both cultured as 2D as well as 3D; however, in spheroids, a 50-fold higher concentration of Tz was needed to observe a significant size reduction (Fig 3C). MCF7 cells did not show significant sensitivity to Tz, neither in 2D nor in 3D cultures. Thus, increased resistance to the cytotoxic effect of Tz appeared to be associated with changes induced by the 3D organization. The 3D architecture of breast cancer cells has a significant impact on the response to chemotherapy [21]. These changes might be the result of the limited drug exposure of the core cells because of poor penetration of Tz, or to limited sensitivity of these cells owing to a reduction in cellular proliferation and/or resistance associated with 3D cell adhesion, as described by other authors [22]. Cells cultured as monolayers are uniformly in contact with the medium and the drugs, opposing to those in 3D, where Tz must diffuse along different and heterogenic gradient of cell subpopulations. It is possible that high intracellular binding and consumption of the drug by peripheral cells in the spheroids limited the toxicity to cells distant from the surface [23].

Cells remaining alive in the spheroids after 15 days of chronic Tz treatment showed full resistance when re-exposed to Tz. Furthermore, the reduction in pAkt observed after 24 h was no longer detected 5 days later, in agreement with Yakes et al, who associated Tz resistance with constitutively active Akt [24]. Our results suggest that resistance to Tz developed in chronically treated BT474 spheroids, is associated with Akt pathway.

To deeply understand the development of Tz resistance enhanced by 3D cell organization, we analyzed the cell subpopulations that remained alive after 15 days treatment. Although downregulation of HER2 receptor has been proposed as one of the mechanisms involved in Tz cytotoxic activity [25], we could not detect differences in HER2 expression between treated and control spheroids. However, the evidence of HER2 downregulation by Tz has been reported mostly from experiments *in vitro* [26], and is not fully supported by *in vivo* findings. [27]. In this aspect, our results appear to be more similar to the *in vivo* response. A decrease in pHER2 expression was detected in treated spheroids, in agreement with other authors who have described inhibition of HER2 phosphorylation upon Tz binding [28]. Our results suggest that HER2 is functional along spheroids growth, and that Tz-acquired resistance could have been developed by a mechanism downstream the receptor.

Wang *et al* have shown that hypoxia induces a HIF-1α-dependent increase in the cyclin dependent kinase inhibitor p27 [29]. In control spheroids, we detected that cells expressing nuclear p27 were located in the same region as nuclear HIF-1α positive cells, both surrounding the necrotic core. It has been reported that in sensitive cells, Tz induces the expression of nuclear p27, driving cell cycle arrest [24]; however, in cells with acquired resistance this translocation is not observed [30]. According to these reports, we can assume that in our 3D model, chronic treatment with Tz induced the sequestration of p27 away from the nucleus associated with the development of a resistant phenotype of the remaining cells.

Taking into account that the inhibitory effect of Tz on spheroids growth could be detected already at 24h, we hypothesized that early changes in cell populations might have occurred. Thus, we analysed the cell cycle distribution and observed that Tz arrested BT474 cells in G0/G1 phase, both in 2D and 3D cultures, without significant effect on MCF7 cells. Untreated BT474 spheroids showed a basal subpopulation of apoptotic cells, not found in monolayer cultures, reduced after Tz treatment. These results suggest that Tz might be rescuing cells from death rather than inducing it; these observations agree with other authors that have also reported that Tz did not induced apoptosis *in vitro* [31,32]. According to our results, and for the first time, we here describe Tz as a downregulator of basal apoptotic cell death.

Studies in breast cancer cells indicate that induction of autophagy plays a protective role in antitumor therapies [33]. Furthermore, in the last years, HER2 overexpression was found to be associated with induction of autophagy [34]. It is known that the basal level of autophagy increases in cancer cells under stress leading to a state of quiescence [35]. Thus, it was reasonable to hypothesize that autophagy could be one of the mechanisms involved in Tz antitumor escape, supported by the reduced cell proliferative capacity (Ki67+) observed in treated BT474 spheroids.

In contrast to monolayers, 3D cultures presented basal autophagy, probably due to the hostile microenvironment. After chronic treatment, spheroids showed higher and uniform LC3 expression compared to control spheroids, in accordance with Tz-resistant cell phenotype described by other authors [36]. The increased sensitivity to Tz found after inhibition of autophagy with 3-MA, suggests that Tz-induced autophagy provided an advantage to cell survival and facilitated the development of acquired resistance. However, in resistant BT474-MR cells, inhibition of autophagy did not increase sensitivity to Tz, indicating that they do not fully relay on autophagy to survive Tz antitumor effect; other mechanisms might be involved. Likewise, autophagy inhibition had no effect on MCF7 cells, which are not responsive to Tz.

Autophagy and apoptosis are fundamental mechanisms in cellular homeostasis and a molecular crosstalk between both pathways has been described [37,38]; thus, we decided to study this link in our model. We detected that the decrease of basal apoptosis exerted by Tz was completely reversed by autophagy inhibition with 3-MA; moreover, Tz was able to induce apoptosis only in these conditions. Our results agree with other authors, who have reported that autophagy inhibition increases cell apoptosis in 2D cultures after addition of different antitumor agents [39]. Therefore, we can conclude that 3D organization confers BT474 cells with a protective microenvironment against the antitumor effect of Tz, not generated in 2D conditions. In the hypoxic core of tumor spheroids, cells might survive eliciting autophagy, which enables them to resist therapy injury. The differences we detected in basal apoptosis between 2D and 3D might reveal the importance of autophagy inhibition in the re-sensitization of cancer cells to Tz. Although other mechanisms could also be involved in tumor evasion to antitumor therapies, authophagy induced by Tz appears to be providing cells the capacity to evolve and develop Tz resistance, thus avoiding tumor cell death.

## References

[1] Press MF, Bernstein L, Thomas PA, Meisner LF, Zhou JY, Ma Y, et al HER-2/neu gene amplification characterized by fluorescence in situ hybridization: poor prognosis in node-negative breast carcinomas. J Clin Oncol. 1997;15: 2894–904. 9256133 10.1200/JCO.1997.15.8.2894

[2] Ghosh R, Narasanna A, Wang SE, Liu S, Chakrabarty A, Balko JM, et al Trastuzumab has preferential activity against breast cancers driven by HER2 homodimers. Cancer Res. 2011;71: 1871–82. 10.1158/0008-5472.CAN-10-1872 21324925 PMC3221734

[3] Junttila TT, Akita RW, Parsons K, Fields C, Lewis Phillips GD, Friedman LS, et al Ligand-independent HER2/HER3/PI3K complex is disrupted by trastuzumab and is effectively inhibited by the PI3K inhibitor GDC-0941. Cancer Cell. Elsevier Ltd; 2009;15: 429–40. 10.1016/j.ccr.2009.03.020 19411071

[4] Musolino A, Naldi N, Bortesi B, Pezzuolo D, Capelletti M, Missale G, et al Immunoglobulin G fragment C receptor polymorphisms and clinical efficacy of trastuzumab-based therapy in patients with HER-2/neu-positive metastatic breast cancer. J Clin Oncol. 2008;26: 1789–96. 10.1200/JCO.2007.14.8957 18347005

[5] Vogel CL, Cobleigh MA, Tripathy D, Gutheil JC, Harris LN, Fehrenbacher L, et al Efficacy and safety of trastuzumab as a single agent in first-line treatment of HER2-overexpressing metastatic breast cancer. J Clin Oncol. 2002;20: 719–26. 11821453 10.1200/JCO.2002.20.3.719

[6] Vu T, Claret FX. Trastuzumab: updated mechanisms of action and resistance in breast cancer. Front Oncol. 2012;2: 62 10.3389/fonc.2012.00062 22720269 PMC3376449

[7] Fiszman GL, Jasnis MA. Molecular Mechanisms of Trastuzumab Resistance in HER2 Overexpressing Breast Cancer. Int J Breast Cancer. 2011;2011: 352182 10.4061/2011/352182 22295219 PMC3262573

[8] Correia AL, Bissell MJ. The tumor microenvironment is a dominant force in multidrug resistance. Drug Resist Updat. Elsevier Ltd; 2012;15: 39–49. 10.1016/j.drup.2012.01.006 22335920 PMC3658318

[9] Muñoz L, Espinosa M, Quintanar-Jurado V, Hidalgo A, Melendez-Zajgla J, Maldonado V. Paradoxial changes in the expression of estrogen receptor alpha in breast cancer multicellular spheroids. Tissue Cell. 2010;42: 334–7. 10.1016/j.tice.2010.07.006 20817241

[10] Trédan O, Galmarini CM, Patel K, Tannock IF. Drug resistance and the solid tumor microenvironment. J Natl Cancer Inst. 2007;99: 1441–54. 10.1093/jnci/djm135 17895480

[11] Klionsky DJ, Emr SD. Autophagy as a regulated pathway of cellular degradation. Science. 2000;290: 1717–21. 11099404 10.1126/science.290.5497.1717 PMC2732363

[12] Negri T, Tarantino E. Chromosome band 17q21 in breast cancer: significant association between beclin 1 loss and HER2/NEU amplification. Genes Chromosomes Cancer. 2010;909: 901–909. 10.1002/gcc 20589936

[13] Maes H, Rubio N, Garg AD, Agostinis P. Autophagy: shaping the tumor microenvironment and therapeutic response. Trends Mol Med. 2013;19: 428–46. 10.1016/j.molmed.2013.04.005 23714574

[14] Del Duca D, Werbowetski T, Del Maestro RF. Spheroid preparation from hanging drops: characterization of a model of brain tumor invasion. J Neurooncol. 2004;67: 295–303. 15164985 10.1023/b:neon.0000024220.07063.70

[15] Koopman G, Reutelingsperger CP, Kuijten GA, Keehnen RM, Pals ST, van Oers MH. Annexin V for flow cytometric detection of phosphatidylserine expression on B cells undergoing apoptosis. Blood. 1994;84: 1415–20. 8068938

[16] Martin HL, Smith L, Tomlinson DC. Multidrug-resistant breast cancer: current perspectives. Breast cancer (Dove Med Press. 2014;6: 1–13. 10.2147/BCTT.S37638 24648765 PMC3929252

[17] Hockel M, Schlenger K, Aral B, Mitze M, Schaffer U, Vaupel P. Association between tumor hypoxia and malignant progression in advanced cancer of the uterine cervix. Cancer Res. 1996;56: 4509–15. 8813149

[18] Berardi DE, Campodónico PB, Díaz Bessone MI, Urtreger AJ, Todaro LB. Autophagy: friend or foe in breast cancer development, progression, and treatment. Int J Breast Cancer. 2011;2011: 595092 10.4061/2011/595092 22295229 PMC3262577

[19] Mizushima N, Yoshimori T. How to interpret LC3 immunoblotting. Autophagy. 2007; 542–545. 17611390 10.4161/auto.4600

[20] Esteve JM, Knecht E. Mechanisms of autophagy and apoptosis: Recent developments in breast cancer cells. World J Biol Chem. 2011;2: 232–8. 10.4331/wjbc.v2.i10.232 22031846 PMC3202127

[21] Pickl M, Ries CH. Comparison of 3D and 2D tumor models reveals enhanced HER2 activation in 3D associated with an increased response to trastuzumab. Oncogene. 2009;28: 461–8. 10.1038/onc.2008.394 18978815

[22] Weigelt B, Lo AT, Park CC, Gray JW, Bissell MJ. HER2 signaling pathway activation and response of breast cancer cells to HER2-targeting agents is dependent strongly on the 3D microenvironment. Breast Cancer Res Treat. 2010;122: 35–43. 10.1007/s10549-009-0502-2 19701706 PMC2935800

[23] Minchinton AI, Tannock IF. Drug penetration in solid tumours. Nat Rev Cancer. 2006;6: 583–92. 10.1038/nrc1893 16862189

[24] Yakes F, Chinratanalab W, Ritter C. Herceptin-induced inhibition of phosphatidylinositol-3 kinase and Akt Is required for antibody-mediated effects on p27, cyclin D1, and antitumor action. Cancer Res. 2002;62: 4132–4141. 12124352

[25] Nahta R, Esteva FJ. HER2 therapy: molecular mechanisms of trastuzumab resistance. Breast Cancer Res. 2006;8: 215 10.1186/bcr1612 17096862 PMC1797036

[26] Bailey TA, Luan H, Clubb RJ, Naramura M, Band V, Raja SM, et al Mechanisms of Trastuzumab resistance in ErbB2-driven breast cancer and newer opportunities to overcome therapy resistance. J Carcinog. 2011;10: 28 10.4103/1477-3163.90442 22190870 PMC3243087

[27] Moulder S, Yakes F, Muthuswamy S. Epidermal growth factor receptor (HER1) tyrosine kinase inhibitor ZD1839 (Iressa) inhibits HER2/neu (erbB2)-overexpressing breast cancer cells in vitro and in vivo. Cancer Res. 2001;1839: 8887–8895. 11751413

[28] Ginestier C, Adélaïde J, Gonçalvès a, Repellini L, Sircoulomb F, Letessier a, et al ERBB2 phosphorylation and trastuzumab sensitivity of breast cancer cell lines. Oncogene. 2007;26: 7163–9. 10.1038/sj.onc.1210528 17525746

[29] Harris AL. Hypoxia—a key regulatory factor in tumour growth. Nat Rev Cancer. 2002;2: 38–47. 10.1038/nrc704 11902584

[30] Kute T, Lack CM, Willingham M, Bishwokama B, Williams H, Barrett K, et al Development of Herceptin resistance in breast cancer cells. Cytometry A. 2004;57: 86–93. 10.1002/cyto.a.10095 14750129

[31] Lee S, Yang W, Lan K, Sellappan S. Enhanced sensitization to taxol-induced apoptosis by herceptin pretreatment in ErbB2-overexpressing breast cancer cells. Cancer Res. 2002; 5703–5710. 12384528

[32] Xia W, Gerard CM, Liu L, Baudson NM, Ory TL, Spector NL. Combining lapatinib (GW572016), a small molecule inhibitor of ErbB1 and ErbB2 tyrosine kinases, with therapeutic anti-ErbB2 antibodies enhances apoptosis of ErbB2-overexpressing breast cancer cells. Oncogene. 2005;24: 6213–21. 10.1038/sj.onc.1208774 16091755

[33] Liu Q, Shi X, Zhou X, Wang D, Wang L, Li C. Effect of autophagy inhibition on cell viability and cell cycle progression in MDA-MB-231 human breast cancer cells. Mol Med Rep. 2014;10: 625–30. 10.3892/mmr.2014.2296 24898397

[34] Vazquez Martin A. Regulatory gene ATG6/BECN1 in ERBB2-positive breast carcinomas: Bypassing ERBB2-induced oncogenic senescence to regulate the efficacy of ERBB2-targeted therapies. Genes Chromosomes Cancer. 2011;290: 284–290. 10.1002/gcc 21319263

[35] Feitelson MA, Arzumanyan A, Kulathinal RJ, Blain SW, Holcombe RF, Mahajna J, et al Sustained proliferation in cancer: Mechanisms and novel therapeutic targets. Semin Cancer Biol. 2015; 10.1016/j.semcancer.2015.02.006 PMC4898971 25892662

[36] Vazquez-Martin A, Oliveras-Ferraros C, Menendez J a. Autophagy facilitates the development of breast cancer resistance to the anti-HER2 monoclonal antibody trastuzumab. PLoS One. 2009;4: e6251 10.1371/journal.pone.0006251 19606230 PMC2708925

[37] Zhu X, Wu L, Qiao H, Han T, Chen S, Liu X, et al Autophagy stimulates apoptosis in HER2-overexpressing breast cancers treated by lapatinib. J Cell Biochem. 2013;114: 2643–53. 10.1002/jcb.24611 23794518

[38] Han J, Hou W, Lu C, Goldstein L a, Stolz DB, Watkins SC, et al Interaction between Her2 and Beclin-1 proteins underlies a new mechanism of reciprocal regulation. J Biol Chem. 2013;288: 20315–25. 10.1074/jbc.M113.461350 23703612 PMC3711298

[39] Choi C, Jung Y, Oh S. Autophagy induction by Capsaicin in malignant human breast cells is modulated by p38 and extracellular signal-regulated mitogen-activated protein kinases and retards cell death by suppressing endoplasmic reticulum stress-mediated. Mol Pharmacol. 2010;1: 114–125. 10.1124/mol.110.063495 20371669

